# Post-operative and long-term outcomes in dialysis and non-dialysis patients undergoing on-pump and off-pump CABG: a nationwide cohort analysis

**DOI:** 10.1186/s13019-025-03615-3

**Published:** 2025-10-22

**Authors:** Chia-Hsun Lin, Chung-Kuan Wu, Vy-Khanh Nguyen, Chien-Wei Chuang, Mingchih Chen

**Affiliations:** 1 Division of Cardiovascular Surgery, Department of Surgery, Shin Kong Wu Ho-Su Memorial Hospital, Taipei, Taiwan; 2 School of Medicine, Fu-Jen Catholic University, New Taipei, Taiwan; 3 Division of Nephrology, Department of Internal Medicine and Division of Digital Informatics Management, Department of Digital Medicine, Shin-Kong Wu Ho-Su Memorial Hospital, 95, Wen-Chang Rd, Shih-Lin, Taipei 111, Taipei, Taiwan; 4 College of Management, School of Health Care Administration, Taipei Medical University, Taipei, Taiwan; 5 AI Development Center, Fu Jen Catholic University, New Taipei City, Taiwan; 6 Graduate Institute of Business Administration, College of Management, Fu Jen Catholic University, No.510, Zhongzheng Rd., Xinzhuang Dist, New Taipei City, 242 Taiwan

**Keywords:** Coronary artery bypass grafting, On-pump, Off-pump, Dialysis, Myocardial infarction, Percutaneous coronary intervention, Mortality

## Abstract

**Background:**

Coronary artery bypass grafting (CABG) is commonly recommended for patients with severe coronary artery disease (CAD). However, the current literature lacks consensus on whether on-pump or off-pump CABG provides superior outcomes between dialysis and non-dialysis patients.

**Methods:**

This nationwide retrospective cohort study analysed the demographic and comorbid data of 31,016 participants in Taiwan between January 1, 2006 and December 31, 2015. CAD patients who had undergone CABG were stratified by presence of dialysis and the procedure type into the following four groups: non-dialysis, on-pump; non-dialysis, off-pump; dialysis, on-pump; and dialysis, off pump. Study outcomes included postoperative complications and long-term follow-up.

**Results:**

Non-dialysis on-pump CABG patients experienced worse postoperative outcomes including higher mortality, longer hospital stays, and increased mechanical ventilation use, compared to non-dialysis off-pump CABG patients. Similar trends were observed between the dialysis on-pump CABG and off-pump CABG groups. Cox regression analysis revealed a lower risk of myocardial infarction (MI) and percutaneous coronary intervention (PCI) but higher mortality among non-dialysis on-pump CABG patients (hazard ratio [HR], 0.911, 0.828, and 1.530; 95% confidence interval [CI], 0.850–0.977, 0.761–0.901, and 1.445–1.621; respectively] compared to non-dialysis off-pump CABG patients. In the dialysis population, on-pump CABG patients showed a higher risk of MI and mortality but lower PCI risk than did dialysis off-pump patients (HR, 1.044, 1.262, and 0.724; 95% CI, 0.824–1.322, 1.107–1.439, and 0.582–0.902; respectively). Five-year Kaplan-Meier analysis revealed similar trends.

**Conclusions:**

Off-pump CABG was generally associated with better mortality outcomes in both non-dialysis and dialysis populations.

**Supplementary Information:**

The online version contains supplementary material available at 10.1186/s13019-025-03615-3.

## Background

Coronary artery bypass grafting (CABG) is a commonly-performed myocardial revascularization surgery that uses venous or arterial grafts to bypass atheromatous blockages in coronary arteries, restoring the blood flow to ischemic myocardium [1]. Of the patients hospitalized for heart failure in Taiwan, 0.81% received CABG intervention [2]. The grafts are typically harvested from the left internal mammary artery or saphenous vein of the lower extremities, and occasionally from the right internal mammary artery depending on the patient’s anatomy and location of the occluded coronary arteries [3].

The American Heart Association recommends CABG for stable ischemic heart disease in cases of significant left main coronary artery stenosis, multivessel coronary artery disease (CAD) with left ventricular systolic dysfunction with ejection fraction less than 50%, or triple-vessel CAD (significant stenosis in 3 major coronary arteries with or without proximal left anterior descending artery [4, 5]. CABG surgery can be divided into two types: off-pump and on-pump, distinguished according to the use of cardiopulmonary bypass and cardiac arrest [6].

Off-pump CABG was developed to mitigate certain perioperative complications associated with the on-pump technique, including myonecrosis during aortic occlusion, cerebrovascular accidents, renal dysfunction and systemic inflammatory response syndrome [7]. Surgeons with extensive experience often prefer off-pump CABG, especially for patients with severe disease of the ascending aorta, while the on-pump method is typically used for patients with hemodynamic compromise [4]. Between 2002 and 2011, the prevalence of off-pump CABG in Taiwan was approximately 30% of all CABG procedures, which is significantly lower than that in Japan but higher than in Western countries [8, 9]. A meta-analysis comparing off-pump CABG to on-pump CABG found that off-pump surgery had significantly lower rates of mortality and major cardiovascular events than did on-pump CABG [10].

Dialysis patients are at higher risks of CAD and cardiovascular death than are non-dialysis patients [11]. In addition, hemodialysis patients who underwent CABG experience greater post-operative complications including prolonged ventilation, pneumonia, stroke, reoperation, longer hospital stays, and poorer survival outcomes than non-dialysis patients [12, 13]. However, the comparative outcomes of on-pump and off-pump CABG in dialysis patients remain underexplored. This study aimed to evaluate nationwide post-operative and long-term outcomes in Taiwan between dialysis and non-dialysis patients undergoing on-pump or off-pump CABG.

## Methods

### Data source and collection

The National Health Insurance Research Database (NHIRD), which is managed by the Health and Welfare Data Science Center under the Ministry of Health and Welfare, Taiwan, contains claims data and detailed information on health services covered by the National Health Insurance (NHI) program. The NHI program in Taiwan was launched in 1985 and currently covers more than 99% of the total population of Taiwan. The NHIRD collects demographic data, ambulatory care, clinic visit records, hospital admissions, operations, prescriptions, disease status, and dialysis history. For this study, we obtained information on all patients with CAD who had CABG registered in the NHIRD, which classifies diseases according to the International Classification of Diseases, 9th and 10th Revisions, Clinical Modification (ICD-9-CM, ICD-10-CM). Extracted data included demographic and clinical data including age, sex, dialysis vintage, comorbid diseases, medications, and short- and long-term outcomes.

### Study outcomes

Post-operative outcomes included post-surgical mortality between days 0 and 21, hospitalization for more than 21 days, the need for mechanical ventilation for more than 2 days, the duration of mechanical ventilator use (days), and myocardial infarction (MI) within 21 days after CABG. The long-term outcomes included MI, primary coronary intervention (PCI) or surgery, and mortality. The patients were followed up for 5 years for long-term outcomes.

### Study design and participants

**Fig. 1:**
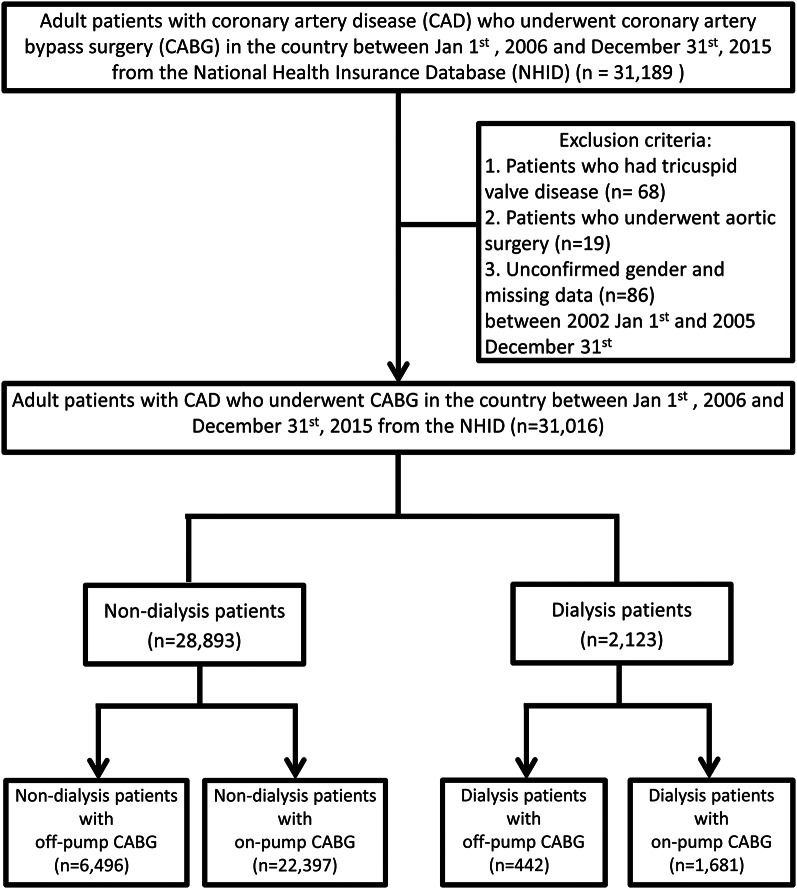
Flowchart of patient selection for study cohort

Data for this nationwide, population-based, retrospective cohort study were collected from the NHIRD. Adult patients with CAD who underwent CABG between January 1, 2006 to December 31, 2015 were included. Of the 31,189 identified patients, those with tricuspid valve disease (*n* = 68), those who underwent aortic surgery (*n* = 19) and those of unconfirmed sex or missing data (*n* = 86) during the period from January 1, 2002 to December 31, 2005 were excluded. The final study cohort comprised 31,016 patients, including 28,893 non-dialysis patients and 2,123 dialysis patients. Each group was further stratified into two subgroups: 22,397 non-dialysis patients who underwent on-pump CABG (72.21%) and 6,496 who underwent off-pump CABG (20.94%); and 1,681 dialysis patients who underwent on-pump CABG (5.42%) and 442 who underwent off-pump CABG (1.43%) (Fig. 1).

### Statistical analysis

The demographic and clinical characteristics of the study cohort were presented as the mean ± standard deviation for continuous variables and as the number (proportion) for categorical variables. Categorical variables were compared using the Chi-square test or Fisher’s exact test when the expected frequency was less than 5. Continuous variables were analysed using the independent student’s t-test or the Mann-Whitney U test, depending on the normality of the data distribution. Additionally, univariate Cox proportional hazard (PH) models were employed to estimate the relative risk (crude hazard ratios [HRs]) for long-term outcomes, followed by multivariate Cox regression analysis. Cumulative incidence rates were calculated using the Nelson-Aalen method, and group data were compared using the log-rank test. Data were extracted and integrated using SAS software version 9.4 (SAS Institute Inc., Cary, NC, USA). Statistical and Cox analyses were performed using R version 4.1.3. Two-sided p-values < 0.05 were considered statistically significant.

## Results

### Patient baseline characteristics

We compared the baseline characteristics of CAD patients according to their dialysis status and the type of CABG they underwent (Table 1). In both the non-dialysis and dialysis groups, patients undergoing on-pump CABG were more frequently female and had a greater number of grafts (*n* ≥ 2) than did patients in the off-pump group. Among the dialysis patients, the mean dialysis vintage did not differ significantly between those undergoing off-pump and on-pump procedures (4.58 ± 3.33 years vs. 4.70 ± 3.40 years, respectively).

**Table 1:** Baseline characteristics of non-dialysis and Dialysis patients undergoing coronary artery bypass grafting (CABG)

	Non-dialysis patients			Dialysis patients		
	Off-pump (n = 6,496)	On-pump (n = 22,397)	***p***	Off-pump (n = 442)	On-pump (n = 1,681)	***p***
Age	64.63 ± 11.28	65.37 ± 11.01	< 0.001^§^	61.86 ± 10.37	62.25 ± 9.71	0.470^§^
Sex_female (%)	1,425 (21.9)	5,224 (23.3)	0.02^†^	125 (28.3)	560 (33.3)	0.050^†^
Graft_amount (*n* = 1)	759 (11.7)	1,860 (8.3)	< 0.001^†^	76 (17.2)	127 (7.6)	< 0.001^†^
Graft_amount (*n* = 2)	913 (14.1)	3,615 (16.1)		77 (17.4)	329 (19.6)	
Graft_amount (*n*>2)	4,824 (74.3)	16,922 (75.6)		289 (65.4)	1,225 (72.9)	
**Comorbidities**,** n (%)**						
DM	3,115 (48.0)	10,583 (47.3)	0.326^†^	324 (73.3)	1,166 (69.4)	0.120^†^
HTN	4,651 (71.6)	15,850 (70.8)	0.2^†^	400 (90.5)	1,478 (87.9)	0.155^†^
Dyslipidemia	3,215 (49.5)	10,400 (46.4)	< 0.001^†^	233 (52.7)	771 (45.9)	0.012^†^
CKD	744 (11.5)	2,632 (11.8)	0.524^†^	442 (100.0)	1,681 (100.0)	N/A
HF	1,757 (27.0)	7,352 (32.8)	< 0.001^†^	246 (55.7)	916 (54.5)	0.701^†^
PAD	527 (8.1)	1,915 (8.6)	0.275^†^	109 (24.7)	350 (20.8)	0.093^†^
CVA	1,253 (19.3)	4,007 (17.9)	0.011^†^	114 (25.8)	343 (20.4)	0.017^†^
Malignancy	273 (4.2)	994 (4.4)	0.434^†^	27 (6.1)	103 (6.1)	1^†^
**Medications**,** n (%)**						
Anti-HTN	5,482 (84.4)	18,758 (83.8)	0.218^†^	352 (79.6)	1290 (76.7)	0.195^†^
CCB	2,573 (39.6)	8,297 (37.0)	< 0.001^†^	195 (44.1)	648 (38.5)	0.038^†^
RASI	3,893 (59.9)	13,356 (59.6)	0.679^†^	199 (45.0)	782 (46.5)	0.611^†^
$\:\alpha\:$-blocker	430 (6.6)	1,411 (6.3)	0.368^†^	41 (9.3)	130 (7.7)	0.336^†^
$\:\beta\:$-blocker	2,907 (44.8)	9,138 (40.8)	< 0.001^†^	177 (40.0)	625 (37.2)	0.294^†^
Vasodilators	125 (1.9)	472 (2.1)	0.387^†^	29 (6.6)	93 (5.5)	0.476^†^
Diuretics	1,722 (26.5)	7,397 (33.0)	< 0.001^†^	97 (21.9)	346 (20.6)	0.574^†^
OAD	3,126 (48.1)	10,734 (47.9)	0.792^†^	282 (63.8)	1,007 (59.9)	0.151^†^
Insulin and analogue	1,119 (17.2)	3,820 (17.1)	0.763^†^	202 (45.7)	689 (41.0)	0.083^†^
Statin	3,299 (50.8)	10,233 (45.7)	< 0.001^†^	159 (36.0)	623 (37.1)	0.714^†^
Fenofibrate	501 (7.7)	1,351 (6.0)	< 0.001^†^	45 (10.2)	157 (9.3)	0.656^†^
Antiplatelet	5,477 (84.3)	17,229 (76.9)	< 0.001^†^	374 (84.6)	1,394 (82.9)	0.438^†^
Antiplatelet on admission	6,221 (95.8)	19,653 (87.7)	< 0.001^†^	411 (93.0)	1,504(89.5)	0.034^†^
Warfarin	126 (1.9)	710 (3.2)	< 0.001^†^	8 (1.8)	39 (2.3)	0.641^†^

DM, diabetes mellitus; HTN, hypertension; HF, heart failure; PAD, peripheral arterial disease; CVA, cerebrovascular accident; CCB, calcium-channel blockers; RASI, renin-angiotensin system inhibitor; OAD, oral antidiabetic drugs

§ Independent sample t-test † Chi-square

Comorbidity analysis showed that the prevalence of cerebrovascular accident and dyslipidemia was significantly lower among on-pump patients in both the dialysis and non-dialysis groups. However, the prevalence of heart failure was significantly higher among non-dialysis on-pump patients compared to their off-pump counterparts.

The use of calcium channel blockers, β-blockers, statins, fenofibrate, and antiplatelets was significantly lower in the on-pump group, while diuretics and warfarin were more commonly used among non-dialysis on-pump patients (*p* < 0.05). In both the dialysis and non-dialysis groups, significantly lower antiplatelet use at admission was observed in the on-pump group (*p* < 0.05).

### Associations between CABG type and long-term outcomes

In both the non-dialysis and dialysis groups, patients who underwent on-pump CABG had a significantly higher prevalence of post-operation mortality between day 0 and 21, hospital stay over 21 days, the need for mechanical ventilation more than 2 days, and longer total ventilation time required compared to those in the off-pump CABG group (Table 2). The prevalence of MI within 21 days of surgery was significantly lower among on-pump than off-pump group in the non-dialysis group only.

**Table 2:** Post-operative and long-term outcomes of non-dialysis and Dialysis patients undergoing coronary artery bypass grafting (CABG)

	Non-dialysis patients			Dialysis patients		
	Off-pump (n = 6,496)	On-pump (n = 22,397)	***p***	Off-pump (n = 442)	On-pump (n = 1,681)	***p***
**Post-operative outcomes**						
Post_OP deaths (D0-D21)	114 (1.8)	1,270 (5.7)	< 0.001^†^	13 (2.9)	207 (12.3)	< 0.001^†^
Hospitalized for > 21 days (%)	1,205 (18.5)	7,851 (35.1)	< 0.001^†^	176 (39.8)	804 (47.8)	0.003^†^
Mechanical ventilator > 2 days	2,255 (34.7)	10,639 (47.5)	< 0.001^†^	184 (41.6)	985 (58.6)	< 0.001^†^
Mechanical ventilator days	4.54 ± 9.82	7.60 ± 16.08	< 0.001^§^	7.28 ± 17.81	10.21 ± 18.68	0.004^§^
MI within 21 days	336 (5.2)	633 (2.8)	< 0.001^†^	12 (2.7)	28 (1.7)	0.212^†^
**Long-term outcomes (5-year follow-up)**						
Mortality	1,389 (21.4)	6,777 (30.3)	< 0.001^†^	276 (62.4)	1149 (68.4)	0.022^†^
MI	1,049 (16.1)	3,398 (15.2)	0.057^†^	86 (19.5)	341 (20.3)	0.749^†^
PCI surgery	720 (11.1)	2,078 (9.3)	< 0.001^†^	108 (24.4)	306 (18.2)	0.004^†^

OP, operation; MI, myocardial infarction; PCI, percutaneous coronary intervention

§ Independent sample t-test †Chi-square test

Regarding long-term outcomes after CABG, the prevalence of PCI was significantly lower among on-pump than off-pump group in both the dialysis and non-dialysis groups (*p* < 0.05), while the mortality rate was significantly higher for on-pump than off-pump patients with or without dialysis (Table 2).

Comparison of baseline characteristics and outcome parameters of single stratifications (i.e. on-pump versus off-pump patients and non-dialysis versus dialysis patients) showed results in line with our core analysis (Supplementary Table 1).

### Association between pump status and the risk of MI, PCI, and mortality among non-dialysis patients

**Fig. 2:**
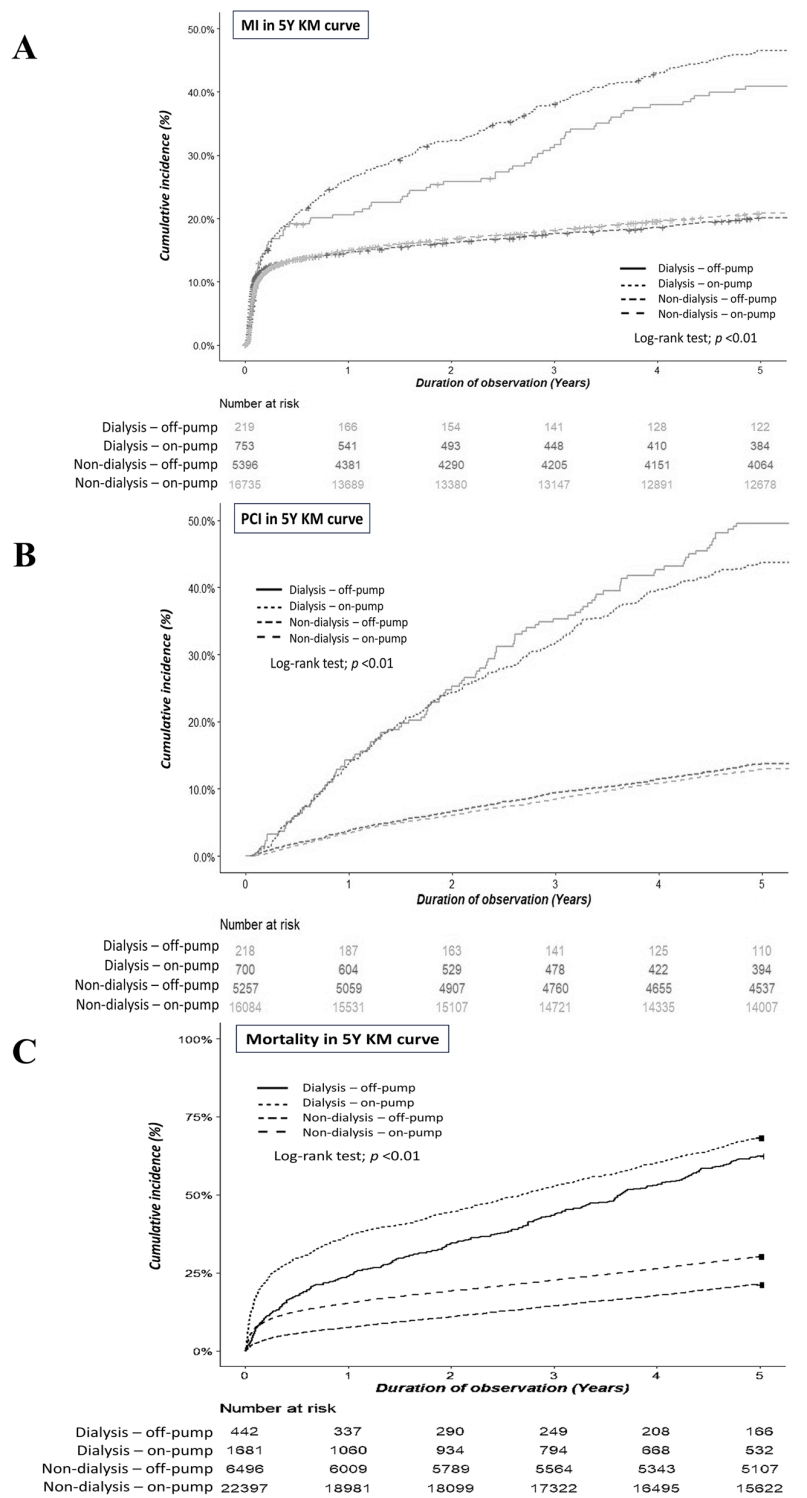
Nelson-Aalen analysis cumulative incidence curves for long-term outcomes of non-dialysis and dialysis patients undergoing on-pump or off-pump CABG, including [**A**] myocardial infarction (MI), [**B**] percutaneous coronary intervention (PCI), and [**C**] mortality

Among non-dialysis patients, cox proportional hazard analysis results showed a significantly lower risk of MI for those undergoing on-pump rather than off-pump CABG (hazard ratio [HR] of 0.911; 95% confidence interval [CI], 0.850–0.977) (Table 3). The adjusted HRs in cox multivariate analysis (models 2 and 3) followed this same trend. During five-year follow-up, Nelson-Aalen analysis revealed a higher cumulative incidence of MI among non-dialysis off-pump CABG patients compared to non-dialysis on-pump patients (Log-rank *p* < 0.001) (Fig. 2A).

**Table 3:** Cox regression model for long-term outcome events of patients underwent coronary artery bypass grafting (CABG)

	Model 1	Model 2	Model 3
	cHR(95%CI)	aHR(95% CI)	aHR(95% CI)
**MI**			
Non-dialysis^§^			
Off-pump	1	1	1
On-pump	0.911 (0.850, 0.977)	0.915 (0.854, 0.980)	0.843 (0.786, 0.904)
Dialysis*			
Off-pump	1	1	1
On-pump	1.044 (0.824, 1.322)	1.050 (0.829, 1.331)	1.045 (0.822, 1.329)
**PCI**			
Non-dialysis^§^			
Off-pump	1	1	1
On-pump	0.828 (0.761, 0.901)	0.834 (0.766, 0.907)	0.853 (0.783, 0.929)
Dialysis*			
Off-pump	1	1	1
On-pump	0.724 (0.582, 0.902)	0.722 (0.580, 0.899)	0.762 (0.609, 0.954)
**Mortality**			
Non-dialysis^§^			
Off-pump	1	1	1
On-pump	1.530 (1.445, 1.621)	1.502 (1.418, 1.591)	1.378 (1.300, 1.460)
Dialysis*			
Off-pump	1	1	1
On-pump	1.262 (1.107, 1.439)	1.252 (1.098, 1.429)	1.274 (1.115, 1.456)

HR, hazard ratio; MI, myocardial infarction; PCI, percutaneous coronary intervention, cHR, crude hazard ratio, aHR, adjusted hazard ratio

**Model 1**, crude model; **Model 2**, adjusted by age and sex; **Model 3**, ^§^Non-dialysis On-pump vs. Off-pump: adjusted by age, sex, dyslipidemia, heart failure, intra-aortic balloon pump, graft, calcium-channel blocker, *B*-blocker, diuretic, statin, fenofibrate, antiplatelet, and warfarin; *Dialysis On-pump vs. Off-pump: adjusted by age, sex, dyslipidemia, graft, calcium-channel blocker

Among non-dialysis patients, univariate regression analysis revealed a significantly lower PCI risk for those undergoing on-pump compared to off-pump CABG compared to those (HR, 0.828; 95% CI, 0.761–0.901) (Table 3). After adjustment by age, sex (model 2), and relevant medications and comorbid diseases (model 3), the results remained the same. Nelson-Aalen analysis of five-year follow-up data showed higher incidence of PCI among non-dialysis off-pump CABG patients compared to non-dialysis on-pump CABG patients (Log-rank test, *p* < 0.001) (Fig. 2B).

Data analysis using the unadjusted model showed a significantly higher mortality risk for non-dialysis on-pump CABG patients compared to off-pump CABG patients (HR, 1.530; 95% CI, 1.445–1.621) (Table 3). This trend remained significant after adjustments made in multivariate models 2 and 3. Additionally, Nelson-Aalen analysis of five-year follow-up data indicated that non-dialysis on-pump CABG patients had a higher mortality rate than did non-dialysis off-pump CABG patients (Log-rank test, *p* < 0.001) (Fig. 2C).

### Association between pump status and the risk of MI, PCI, and mortality among Dialysis patients

Univariate Cox proportional hazard analysis of MI risk among dialysis patients indicated a higher trend risk for those undergoing in dialysis on-pump compared to off-pump CABG (HR, 1.044; 95% CI, 0.824–1.322) (Table 3). Adjusted analysis using multivariate models 2 and model 3 revealed the same trend. Nelson-Aalen analysis of five-year follow-up, data showed a higher cumulative incidence of MI among dialysis on-pump CABG patients compared to dialysis off-pump CABG group (Log-rank test, *p* < 0.001) (Fig. 2A).

Furthermore, the unadjusted model revealed a significantly lower PCI risk among dialysis patients undergoing on-pump CABG compared to off-pump CABG (HR, 0.724; 95% CI, 0.582–0.902) (Table 3). After adjustment by age, sex (model 2), and additional relevant parameters (model 3), the trend remained. Nelson-Aalen analysis of five-year follow-up data on PCI indicated a higher incidence of PCI among dialysis off-pump rather than on-pump CABG (Log-rank test, *p* < 0.001, Fig. 2B).

Among dialysis patients, univariate analysis showed a significantly higher mortality rate among on-pump CABG patients compared to off-pump CABG patients (HR, 1.262; 95% CI, 1.107–1.439) (Table 3). This trend remained significant after adjustments made in multivariate models 2 and 3. Additionally, Nelson-Aalen analysis showed that among dialysis patients, those undergoing on-pump CABG had a higher cumulative mortality rate than those with off-pump CABG (Log-rank test, *p* < 0.001) (Fig. 2C).

Cox regression analysis of single stratification data (i.e. on-pump versus off-pump CABG; dialysis versus non-dialysis patients) showed similar results to the core analysis and were summarized in Supplementary Table 2.

## Discussion

Our study evaluated the postoperative and long-term outcomes of CABG patient cohorts stratified by different combinations of CABG procedural types and dialysis status. Among non-dialysis patients, those who underwent on-pump CABG generally had a lower risk of postoperative MI and the need for PCI but a higher risk of mortality compared to the off-pump group. Among dialysis patients, on-pump CABG was associated with a higher risk of MI and mortality but a lower prevalence of PCI compared to off-pump CABG.

Although our analysis showed generally better postoperative outcomes for non-dialysis patients undergoing off-pump compared to on-pump CABG, the prevalence of MI remained higher in the non-dialysis off-pump CABG group, even in long-term outcomes (Table 2). Furthermore, a higher prevalence of PCI (Table 2) and an increased risk of PCI (Table 3) were observed in non-dialysis off-pump CABG patients compared to their on-pump counterparts. These findings suggest a potential correlation between MI and PCI, where incomplete revascularization associated with off-pump CABG may contribute to a higher risk of future MI or the need for PCI as an additional intervention [14–16].

The lower mortality rate (Table 2) and lower mortality risk observed in the non-dialysis off-pump group during the 5-year follow-up period (Table 3) were consistent with findings from previous high-quality studies by Park et al. and Ogawa et al.^15,16^. The higher survival rate among off-pump patients present a compelling advantage that outweighs the benefits seen in the on-pump group, despite the latter’s lower risk of MI and need for PCI. While off-pump CABG is a promising treatment option for CAD in the non-dialysis population, it remains essential to implement stringent risk management and follow-up strategies to address the elevated risks of MI and PCI associated with this approach.

Trends similar to those observed in non-dialysis patients were evident in the dialysis population, where the on-pump CABG group exhibited poorer postoperative outcomes than did the off-pump CABG group. Although the prevalence of postoperative MI was comparable between the two groups, this finding reflects a potential disadvantage of off-pump CABG due to the lack of myocardial protection provided by cardioplegia [17]. However, our 5-year follow-up data revealed that the prevalence of MI was significantly higher in the dialysis on-pump CABG group than in the off-pump group (Fig. 2A). Additionally, the lower prevalence (Table 2) and reduced risk (Table 3) of PCI observed in dialysis on-pump CABG patients compared to their off-pump counterparts suggest a possible association between off-pump CABG and incomplete revascularization, which contribute to the increased need for subsequent interventions in dialysis patients undergoing off-pump procedures [14–16]. Furthermore, both overall mortality prevalence and risk were lower in dialysis off-pump CABG patients compared to those undergoing on-pump CABG. These findings were consistent with a previous study using data from the United States Renal Data System database, which demonstrated that off-pump surgery was associated with lower overall mortality in dialysis patients compared to on-pump surgery [18].

The use of cardiopulmonary bypass (CPB) in on-pump surgery has been known to trigger a potent systemic inflammatory response syndrome (SIRS) [19]. This response is driven by the contact of blood with the artificial surfaces of the bypass circuit, ischemia-reperfusion injury, and other non-physiological aspects of the procedure. These inflammatory cascades can contribute to a range of postoperative complications and multi-organ dysfunction, including acute kidney injury, respiratory failure, and neurological deficits, all of which are independent predictors of both short- and long-term non-cardiac mortality [20]. The provides a plausible biological mechanism for the increased all-cause mortality observed in the on-pump group, separate from the risk of primary cardiac events such as MI.

In contrast, off-pump CABG avoids the systemic insult of CPB but is technically more demanding, as it is performed on a beating heart [21]. However, this technical complexity has been associated with a higher likelihood of incomplete revascularization, particularly involving vessels on the lateral or posterior walls of the heart [22]. Incomplete revascularization is a well-established risk factor for future adverse cardiac events, including late MI and the need for subsequent percutaneous coronary intervention (PCI) [23]. However, in dialysis patients, on-pump CABG was associated not only with increased mortality but also with a higher risk of MI. We hypothesize that in the population with exceptionally high-risk of cardiovascular events, characterized by chronic inflammation. Systemic inflammatory insults induced by CPB is poorly tolerated on dialysis patients with chronic inflammation, which may outweigh the potential benefits of technically more complete revascularization. The amplified inflammatory stress may precipitate the occurrence of myocardial infarction.

In practice, the choice of surgical approach depends on multiple factors, including patient characteristics and the complexity of their CAD progression, particularly in the context of dialysis. However, several factors may contribute to the perception that dialysis on-pump patients experience worse outcomes than do those who are off-pump. First, dialysis patients often have compromised cardiovascular status, making them more vulnerable to hemodynamic instability during on-pump surgery [24]. The use of cardiopulmonary bypass and aortic cross-clamping can lead to significant blood pressure fluctuations, adversely affecting cardiac function. Second, systemic inflammation, malnutrition, and dialysis-related risks such as uremia-induced platelet dysfunction increase the likelihood of bleeding complications and hinder recovery [25]. These factors exacerbate the challenges associated with on-pump surgery. Finally, chronic dialysis patients who are older and have severe CAD are a high-risk population. For these patients, off-pump CABG may offer advantages, particularly in reducing operative mortality and cardiopulmonary complications [26, 27].

### Limitations

Our study has several limitations due to its observational, retrospective design, analysis of the cohort selected from the national health database reveals potential associations rather than establishing causality. Additionally, data for on-pump CABG procedures were not further subcategorized into on-pump beating heart surgery and on-pump arrested surgery (on-pump non-beating heart surgery), leaving the proportions of these subtypes undefined. Furthermore, the study was limited by the presence of confounding variables inherent to the use of a national administrative database, which lacks key clinical and surgical-specific data. First, the severity of coronary artery disease (CAD) could not be assessed due to the unavailability of anatomical details such as the SYNTAX score or degree of vessel calcification. Second, left ventricular ejection fraction (LVEF), a well-established predictor of mortality, was not captured in the dataset. Third, patient’s frailty and functional status—both strong predictors of postoperative morbidity and mortality—are not captured in claims data. Fourth, the aforementioned data may have influenced the choice of surgical procedures, and surgical expertise and institutional volume could also have impacted outcomes. For example, off-pump CABG is technically more demanding than conventional on-pump surgery [21, 28]. The outcomes of off-pump CABG are known to be operator-dependent, with high-volume, experienced surgeons achieving significantly better results than those with less experience [29]. Some studies have even suggested that the inability of large randomized trials to demonstrate a clear benefit for off-pump CABG may be attributable to the inclusion of surgeons with varying levels of expertise [30].

Fifth, while the proportion of non-dialysis patients with chronic kidney disease was estimated, the NHI database did not specify the chronic kidney disease stages for non-dialysis participants. This lack of granularity may have influenced the subgroup analyses and interpretation of the findings. In addition, dialysis patients were not classified into hemodialysis and peritoneal dialysis, which might have different outcomes. Finally, as the study was based on Taiwan’s national health database, the majority of the cohort was Asian. This limitation could introduce a racial confounding factor, limiting the generalizability of our findings. Future extrapolation of these results to other racial groups may require additional adjustments and parameter calibrations.

## Conclusions

Non-dialysis patients undergoing off-pump CABG experienced lower long-term mortality but had worse long-term outcomes regarding MI and PCI compared to those undergoing on-pump CABG. Among dialysis patients, those undergoing on-pump CABG had a significantly higher risk profile, with worse postoperative and long-term outcomes, including higher rates of MI, mortality, hospitalization, and medical ventilation use, compared to the off-pump group. Further well-conducted studies with refined methodologies and controlled randomization are needed, particularly to explore optimal care strategies for dialysis patients with severe CAD.

## Supplementary Information

Supplementary Material 1.

## Data Availability

No datasets were generated or analysed during the current study.

## Abbreviations

CABG: Coronary artery bypass grafting
CAD: Coronary artery disease
NHIRD: Taiwan National health insurance research database
HR: Hazard ratio
CI: Confidence interval
ICD9-CM: International classification of diseases,9th revisions, clinical modification
ICD10-CM: International classification of diseases,10th revisions, clinical modification
MI: Myocardial infarction
PCI: Percutaneous coronary intervention
