# Supplementary material for: Post-operative and long-term outcomes in dialysis and non-dialysis patients undergoing on-pump and off-pump CABG: a nationwide cohort analysis
Source: J Cardiothorac Surg. 2025 Oct 22;20:379. doi: 10.1186/s13019-025-03615-3 (PMC12542387; doi:10.1186/s13019-025-03615-3)

**Supplementary Table 1.** Baseline characteristics and outcomes of patients who underwent coronary artery bypass grafting (CABG) according to pump and dialysis status

|  | **Off-pump**  (n = 6938) | **On-pump**  (n = 24078) | ***p*** | **No dialysis**  (n = 28893) | **Dialysis**  (n = 2123) | ***p*** |
| --- | --- | --- | --- | --- | --- | --- |
| Age | 64.46 ± 11.25 | 65.15 ± 10.95 | <0.001^§^ | 65.20 ± 11.08 | 62.17 ± 9.85 | <0.001^§^ |
| Sex, female (%) | 1550 (22.3) | 5784 (24.0) | 0.004^†^ | 6649 (23.0) | 685 (32.3) | <0.001^†^ |
| Graft amount (n = 1) | 835 (12.0) | 1987 (8.3) | <0.001^†^ | 2619 (9.10) | 203 (9.6) | 0.001^†^ |
| Graft amount (n = 2) | 990 (14.3) | 3944 (16.4) |  | 4528 (15.7) | 406 (19.1) |  |
| Graft amount (> 2) | 5113 (73.7) | 18147 (75.4) |  | 21746 (75.3) | 1514 (71.3) |  |
| **Comorbidities** (%) | | | | | | |
| DM | 3439 (49.6) | 11749 (48.8) | 0.257^†^ | 13698 (47.4) | 1490 (70.2) | <0.001^†^ |
| HTN | 5051 (72.8) | 17328 (72.0) | 0.171^†^ | 20501 (71.0) | 1878 (88.5) | <0.001^†^ |
| Dyslipidemia | 3448 (49.7) | 11171 (46.4) | <0.001^†^ | 13525 (46.8) | 1004 (47.3) | 0.668^†^ |
| CKD | 1186 (17.1) | 4313 (17.9) | 0.116^†^ | 3376 (11.7) | 2123 (100.0) | n/a |
| HF | 2003 (28.9) | 8268 (34.3) | <0.001^†^ | 9109 (31.5) | 1162 (54.7) | <0.001^†^ |
| PAD | 636 (9.2) | 2265 (9.4) | 0.349^†^ | 2442 (8.5) | 459 (21.6) | <0.001^†^ |
| CVA | 1367 (19.7) | 4350 (18.1) | 0.002^†^ | 5260 (18.2) | 457 (21.5) | 0.001^†^ |
| Malignancy | 300 (4.3) | 1097 (4.6) | 0.412^†^ | 1267 (4.4) | 130 (6.1) | 0.001^†^ |
| **Medications** (%) | | | | | | |
| Anti-HTN | 5834(84.1) | 20048(83.3) | 0.107^†^ | 24240 (11.08) | 1642 (9.85) | <0.001^†^ |
| CCB | 2768 (39.9) | 8945 (37.2) | 0.001^†^ | 10870 (37.6) | 843 (39.7) | 0.056^†^ |
| RASI | 4092 (59.0) | 14138 (58.7) | 0.696^†^ | 17249 (59.7) | 981 (46.2) | <0.001^†^ |
| $\alpha$-blocker | 471 (6.8) | 1541 (6.4) | 0.247^†^ | 1841 (6.4) | 171 (8.1) | 0.002^†^ |
| $\beta$-blocker | 3084 (44.5) | 9763 (40.5) | <0.001^†^ | 12045 (41.7) | 802 (37.8) | 0.001^†^ |
| Vasodilators | 154 (2.2) | 565 (2.3) | 0.536^†^ | 597 (2.1) | 122 (5.7) | <0.001^†^ |
| Diuretics | 1819 (26.2) | 7743 (32.2) | <0.001^†^ | 9119 (31.6) | 443 (20.9) | <0.001^†^ |
| OAD | 3408 (49.1) | 11741 (48.8) | 0.599^†^ | 13860 (48.0) | 1289 (60.7) | <0.001^†^ |
| Insulin and analogue | 1321 (19.0) | 4509 (18.7) | 0.556^†^ | 4939 (17.1) | 891 (42.0) | <0.001^†^ |
| Statin | 3458 (49.8) | 10856 (45.1) | <0.001^†^ | 13532 (46.8) | 782 (36.8) | <0.001^†^ |
| Fenofibrate | 546 (7.9) | 1508 (6.3) | <0.001^†^ | 1852 (6.4) | 202 (9.5) | <0.001^†^ |
| Antiplatelet | 5851 (84.3) | 18623 (77.3) | <0.001^†^ | 22706 (78.6) | 1768 (83.3) | <0.001^†^ |
| Post-antiplatelets | 6632 (95.6) | 21157 (87.9) | <0.001^†^ | 25874 (89.6) | 1915 (90.2) | 0.343^†^ |
| Warfarin | 134 (1.9) | 749 (3.1) | <0.001^†^ | 836 (2.9) | 47 (2.2) | 0.069^†^ |
| **Post-operative outcomes** | | | | | | |
| Death (D0_D21) | 127 (1.8) | 1477 (6.1) | <0.001^†^ | 1384 (4.8) | 220 (10.4) | <0.001^†^ |
| Hospitalization > 21 days (%) | 1381 (19.9) | 8655 (35.9) | <0.001^†^ | 9056 (31.3) | 980 (46.2) | <0.001^†^ |
| Mechanical ventilation > 2 days | 2439 (35.2) | 11624 (48.3) | <0.001^†^ | 12894 (44.6) | 1169 (55.1) | <0.001^†^ |
| Mechanical ventilation days (n) | 1.95 ± 10.7 | 6.71 ± 16.3 | <0.001^§^ | 5.34 ± 15.1 | 9.86 ± 18.4 | <0.001^§^ |
| MI within 21 days | 348 (5.0) | 661 (2.7) | <0.001^†^ | 969 (3.4) | 40 (1.9) | 0.001^†^ |
| **Long-term outcomes (5-year follow-up)** | | | | | | |
| Mortality | 1665 (24.0) | 7926 (32.9) | <0.001^†^ | 8166 (28.3) | 1425 (67.1) | <0.001^†^ |
| MI | 1135 (16.4) | 3739 (15.5) | 0.094^†^ | 4447 (15.4) | 427 (20.1) | <0.001^†^ |
| PCI or surgery | 828 (11.9) | 2384 (9.9) | <0.001^†^ | 2798 (9.7) | 414 (19.5) | <0.001^†^ |

Abbreviations: DM, diabetes mellitus; HTN, hypertension; HF, heart failure; PAD, peripheral arterial disease; CVA, cerebrovascular accident; CCB, calcium-channel blockers; RASI, renin-angiotensin system inhibitor; OAD, oral antidiabetic drugs; OP, operation; MI, myocardial infarction; PCI, percutaneous coronary intervention.

^§^Independent sample t-test; ^†^Chi-square test

**Supplementary Table 2**. Cox regression analysis of long-term outcomes of patients who underwent coronary artery bypass grafting (CABG)

|  | **Model 1** | **Model 2** | **Model 3** |
| --- | --- | --- | --- |
|  | HR (95% CI) | aHR (95% CI) | aHR (95% CI) |
| **MI** | | | |
| ^§^Non-dialysis | 1 | 1 | 1 |
| ^§^Dialysis | 1.295 (1.172–1.43) | 1.263 (1.143–1.396) | 1.263 (1.138–1.402) |
| *Off-pump | 1 | 1 | 1 |
| *On-pump | 0.922 (0.863–0.985) | 0.926 (0.867–0.990) | 0.861 (0.805–0.920) |
| **PCI** | | | |
| ^§^Non-dialysis | 1 | 1 | 1 |
| ^§^Dialysis | 2.144 (1.934–2.377) | 2.027 (1.826–2.249) | 1.960 (1.754–2.189) |
| *Off-pump | 1 | 1 | 1 |
| *On-pump | 0.820 (0.758–0.888) | 0.825 (0.762–0.893) | 0.849 (0.784–0.919) |
| **Mortality** | | | |
| ^§^Non-dialysis | 1 | 1 | 1 |
| ^§^Dialysis | 3.179 (3.005–3.364) | 3.918 (3.700–4.150) | 3.218 (3.027–3.421) |
| *Off-pump | 1 | 1 | 1 |
| *On-pump | 1.490 (1.414–1.571) | 1.464 (1.389–1.544) | 1.361 (1.290–1.435) |

HR, hazard ratio; MI, myocardial infarction; PCI, percutaneous coronary intervention, cHR, crude hazard ratio, aHR, adjusted hazard ratio

**Model 2** Adjusted by age and sex

**Model 3** ^§^Dialysis vs Non-dialysis adjusted by age + sex + dyslipidemia + heart failure + intra-aortic balloon pump+ graft + ACEI_ARB + β-blocker + α-blocker + Vasodilator + Diuretic + oral antidiabetic drugs + insulin + statin + fenofibrate + antiplatelet

*On-pump vs Off-pump adjusted by age + sex + dyslipidemia + heart failure + intra-aortic balloon pump + graft + calcium channel blocker + β-blocker + diuretic + Statin + Fenofibrate + antiplatelet + warfarin

**Supplementary Figure 1**. Nelson–Aalen cumulative incidence curves comparing long-term outcomes after CABG including myocardial infarction (MI) [**A**] on-pump versus off-pump group, [**B**] dialysis versus non-dialysis group; percutaneous coronary intervention (PCI) [**C**] on-pump versus off-pump group, [**D**] dialysis versus non-dialysis group; and mortality [**E**] on-pump versus off-pump group, [**F**] dialysis versus non-dialysis group.


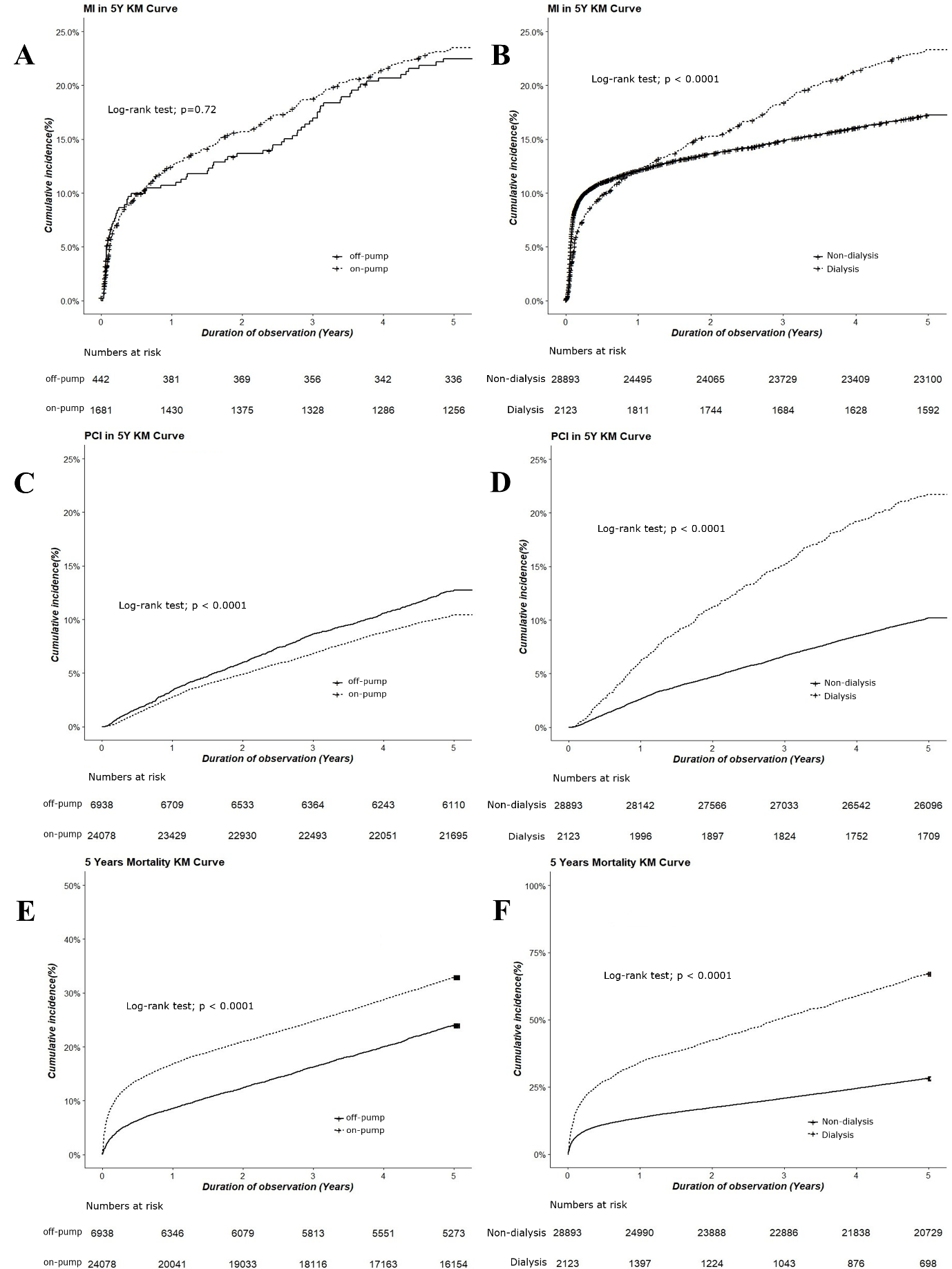

Supplement: Supplementary file 1 — Supplementary Material 1. [file 13019_2025_3615_MOESM1_ESM.docx]
